# Retraction: Effect of controlled and uncontrolled cooling rate on motility parameters of cryopreserved ram spermatozoa

**DOI:** 10.1186/1756-0500-5-319

**Published:** 2012-06-21

**Authors:** Iraj Ashrafi, Hamid Kohram, Hamid Naijan, Majid Bahreini, Hamid Mirzakhani

**Affiliations:** 1 Young Researchers Club, Science and Research Branch, Islamic Azad University, Tehran, Iran; 2 Department of Animal Science, Faculty College of Agriculture and Natural Resources, University of Tehran, Karaj, Iran; 3 Department of Clinical Sciences, Faculty of Veterinary Medicine, Shahid Chamran University, Ahvaz, Iran; 4 Animal Breeding Center of IRAN, Karaj, Iran

## Retraction

This article [1] has been retracted at the request of the Editor. Although the authors withdrew their submission in order to publish elsewhere, the article was subsequently transmitted to the journal’s production department which resulted in it being published in error.
